# Data reduction when aggregating information about harms associated with medical interventions

**DOI:** 10.1136/bmjment-2024-301025

**Published:** 2024-03-21

**Authors:** Edoardo Giuseppe Ostinelli, Toshi A Furukawa

**Affiliations:** 1 Department of Psychiatry, University of Oxford, Oxford, UK; 2 Oxford Health NHS Foundation Trust, Oxford, UK; 3 Oxford Precision Psychiatry Lab, NIHR Oxford Health Biomedical Research Centre, Oxford, UK; 4 Department of Health Promotion and Human Behaviour, School of Public Health in the Graduate School of Medicine, Kyoto University, Kyoto, Japan

**Keywords:** Depression & mood disorders

**Figure 1:**
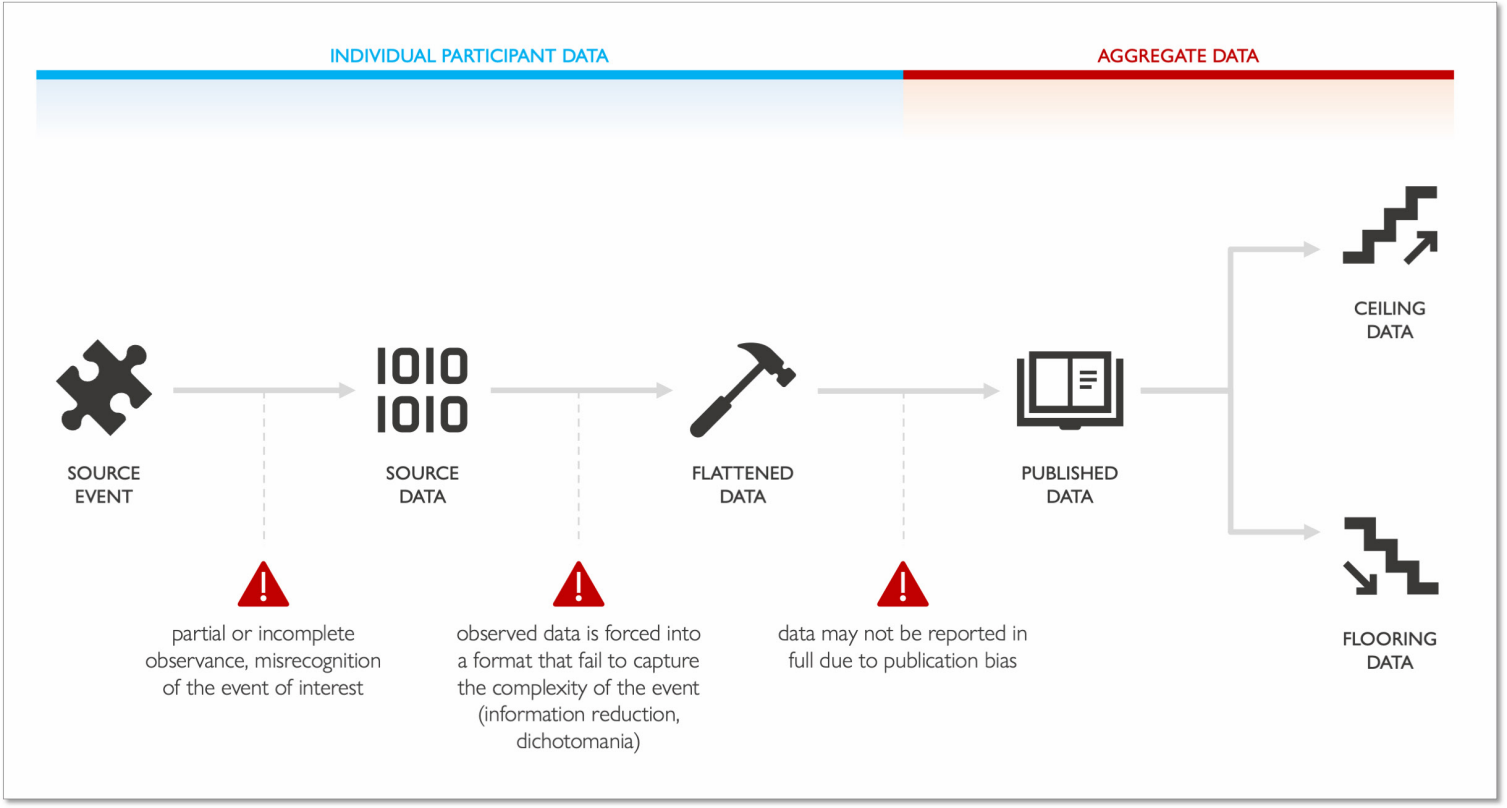
Chain of data loss or reduction. Of the available information on an event of interest (source event), only what is observed can be captured as source data. According to study-specific requirements, data may be flattened into distinct formats (data flattening), subsequently reported as an aggregate value (published data). Previous data loss introduces ambiguity in their downstream interpretation (ceiling, flooring).

In interpreting and aggregating data in published reports, readers and authors must be aware that some data loss and transformation are inevitable in the process (figure 1).^1^ Kamp and colleagues recently examined the beneficial and adverse event (AE) profiles of tricyclic antidepressants in a systematic review of available evidence from randomised controlled trials. The authors identified 103 trials randomising 10 590 participants, concluding that in the short term these medications may reduce depressive symptoms (mean difference on the 17-item Hamilton Rating Scale for Depression of −3.77, 95% CIs −5.91 to –1.63; 17 studies; low certainty of evidence) and increase the chances of ‘serious AEs’ (SAEs) (OR 2.78, 95% CI 2.18 to 3.55; 35 trials; very low certainty of evidence) compared with placebo.^2^

The International Council for Harmonisation of Technical Requirements for Pharmaceuticals for Human Use (ICH), the European Medicines Agency, and the Food and Drug Administration define AEs as ‘any untoward medical occurrence in a patient or clinical investigation subject administered a pharmaceutical product and which does not necessarily have to have a causal relationship with this treatment’. An AE is considered serious and thus cause regulatory implications when it ‘results in death, is life-threatening, requires inpatient hospitalisation or prolongation of existing hospitalisation, results in persistent or significant disability/incapacity, or is a congenital anomaly/birth defect’, with each criterion being evaluated at a patient and event level.^3–5^ For instance, ‘the term *life-threatening* in the definition of *serious* refers to an event in which the patient was at risk of death at the time of the event, rather than an event which hypothetically might have caused death if it were more severe’.

In their systematic review, Kamp and colleagues applied their own judgement in categorising specific AEs as non-serious or serious, ultimately consisting in a worst-case scenario when severity details were considered inadequately reported by the original authors.^2^ For instance, ‘taste alteration/perversion’ was considered an SAE occurring in 26 out of 677 participants enrolled in four studies (figure S18, Kamp and colleagues).^2^ Moreover, they had access only to aggregate data to evaluate the seriousness of AEs. Not all the AEs that, on average, are associated to additional care are SAEs at an individual level (eg, not all individuals experiencing blurred vision will require hospitalisation or will be in a life-threatening condition; figure S16, Kamp and colleagues).^2^ The widely accepted definition of SAE appeared in the mid-1990s.^3^ As 11 out of the 103 studies contributing to the primary outcomes were published after 2000, it is expected that the original investigators did not report the exact numbers of SAEs as currently understood.

When observed clinical information (source event) is translated into source data at the collection site, how data are measured will set implications downstream (figure 1). This is where data flattening can occur, a process where data are simplified via reduction of their number of dimensions (eg, instead of measuring a variable as continuous, it is categorised into an ordinal variable or dichotomised). This may happen voluntarily to reduce the amount of information stored or to avoid collecting data that are considered not relevant. After data are flattened, restoration of lost information is not possible, with imputation being the only possible solution.^6^ External researchers are limited to flattened aggregate data reported by original authors (published data). When seriousness of AEs is not clearly reported, researchers can (1) renounce to use that data, (2) consider all the events as non-serious (flooring, best-case scenario) or (3) consider all as serious (ceiling) based on the average outcome (ie, a specific AE usually results in hospitalisation) or the worst outcome (ie, a specific AE may worsen and result in hospitalisation, worst-case scenario). Any of these assumptions might generate deviations from the truth and should be carefully examined and discussed.^7^

It is truly important to understand the absolute and relative frequencies of AEs and potential harms associated with medical interventions.^8^ We applaud the authors’ efforts towards this goal. Access to individual participant data can overcome reporting bias but, to limit information loss, what data should be collected and in which format should follow rigorous criteria widely established across the regulatory and scientific communities (standardisation). This is increasingly important given their emerging role in shared decision-making processes and patient decision aids aimed at identifying who may be at higher risk of experiencing harms.^9^

For the very same reason, it is essential to rely on a lingua franca of research on medical interventions when referring to aggregate data on harms.^9^ Additionally, uniformity on characterisation and format of safety data would translate into dataset harmonisation across studies, countries and sponsors, introducing several benefits^10^: federated analyses would bypass data sharing agreement while preserving individual patient’s privacy, increasing access to data and maximising transparency^11^; linking harmonised data to pharmacovigilance repositories would be facilitated, allowing real-time contribution of multiple data sources into a synchronous environment.
